# Co‐production with young people with developmental language disorder: Developing adapted materials for cognitive behavioral therapy

**DOI:** 10.1002/jcv2.70121

**Published:** 2026-04-21

**Authors:** Isabella Metcalfe, Courtenay Norbury, The DLD Young Person and Professionals Advisory Group, Rachel Beaumont, Siouxsie Webster, Rachel Hardy, Daniel Hardy, Jo Saul, Freddie Saul, Ben Lightfoot, Izumi Kershaw, Isaiah Efe, Katie DeBonis, Sarah Griffiths

**Affiliations:** ^1^ Department of Clinical Educational and Health Psychology University College London London UK

**Keywords:** anxiety, co‐production, cognitive behavioral therapy, depression, developmental language disorder, psychoeducational materials, young people

## Abstract

**Background:**

Young people with Developmental Language Disorder (DLD) are at increased risk of mental health difficulties but often face barriers to accessing traditional talking therapies such as Cognitive Behavioral Therapy (CBT). Co‐production offers a way to create more accessible interventions by involving those with lived experience as well as practitioners. However, co‐producing with young people with DLD remains rare due to high language demands and inaccessible formats.

**Aim:**

To explore how experienced‐based co‐design (EBCD) methodologies could be shaped to include young people with DLD, in the context of adapting a set of psychoeducational videos for depression and anxiety.

**Methods:**

The UK‐based project team consisted of 4 adolescents with DLD, their parents, and six Educational Mental Health Practitioners and 3 academic researchers. A five‐stage process included recruiting team members, identifying therapy adaptations, selecting key psychoeducational concepts, developing scripts and videos, and reflecting on the process. Meetings were adapted to maximize accessibility through simplified language, visual aids, pre‐meeting preparation videos, asynchronous tasks, and post‐meeting summaries.

**Results:**

Ten adapted psychoeducational videos were co‐produced. Key adaptations included slowed pacing, simplified vocabulary and concrete examples. Team members reported that the process felt inclusive and flexible, and that the final materials were more meaningful and relevant. Challenges included balancing parental input with young people's voices, managing conflicting feedback, and ensuring tasks were not overly demanding. Researcher reflexivity and flexibility was critical to maintaining engagement.

**Conclusion:**

This project demonstrates that, with appropriate adaptation, co‐production with young people with DLD and practitioners is both feasible and valuable. The process resulted in more accessible therapeutic materials and offers a starting point for further research involvement.

## Key Points

**What's known?**

- Young people with Developmental Language Disorder (DLD) are at higher risk of mental health issues and find traditional “talking therapies” challenging.
- Co‐production can help make interventions more accessible by involving people with lived experience, but current methods inadvertently exclude young people with DLD.

**What's new?**

- This project adapted EBCD methodologies to include young people with DLD. We detailed the process, specific adaptations, successes and challenges.
- We co‐produced a set of tailored psychoeducational videos.

**What's relevant?**

- The process demonstrated it's possible to successfully integrate the perspectives of young people with DLD and practitioners into co‐production, creating valuable psychoeducational resources and a guide for future researchers to follow.

## INTRODUCTION

Research, healthcare and social policy development has long been perceived as a process driven by professionals, with little input from those who are directly impacted by the outcomes. However, in recent years, there has been a shift towards “co‐production”—a collaborative approach whereby key stakeholders' perspectives and experiences are integrated into the process (Hassett et al., 2024). One stakeholder group that may particularly benefit from co‐production, but have been largely understudied in this context, is young people (adolescents aged 10–19 years; WHO, 2001) with Developmental Language Disorder (DLD). DLD is a lifelong condition characterized by difficulties understanding and using spoken language, with no known biomedical etiology (Bishop et al., 2017). The inclusion of young people with DLD in co‐production is novel for two key reasons. First, there are no previous examples of including young people with DLD in the research process (Hassett et al., 2024). Second, language disorders represent a significant barrier to inclusion in co‐production, as this process typically relies on verbal communication (Griffiths et al., 2025). As such, the co‐production process itself may need to be adapted to ensure the meaningful involvement of young people with DLD. This paper provides an illustration of how young people with DLD can be included in the research process, by describing a project that co‐produced a set of adapted psychoeducational materials for depression and anxiety.

### Why do we need adapted mental health materials for young people with DLD?

DLD is a lifelong condition, with language‐related difficulties first appearing in early childhood and persisting through adolescence and into adulthood, each stage bringing its own challenges (Norbury et al., 2024). Here we focus on adolescents with DLD aged 10–19 (WHO, 2001), a developmental period characterized by new social networks, hormonal changes and increased vulnerability to mental health issues (Blakemore, 2019). Young people with DLD are twice as likely to experience internalizing and externalizing problems than those with typical language development (Yew & O’Kearney, 2013). In addition, language disorder is strongly linked with emotional behavioral disorders (Hollo et al., 2014), anxiety (Cohen et al., 2013) and depression (Conti‐Ramsden & Botting, 2008). It is therefore vital that young people with DLD can access effective mental health interventions.

Young people with DLD may struggle to engage in traditional evidence‐based mental health interventions given they are typically language intensive (“talking therapies” by name), use complex vocabulary and grammatical structures, and examine abstract concepts like core beliefs, catastrophising and emotional regulation (Hill et al., 2025). White et al. (2015) found that verbal ability significantly predicted treatment success (via symptom reduction) following CBT for anxiety in young people with co‐occurring autism and anxiety. Furthermore, O’Keeffe et al. (2018) found that young people with depression were more likely to drop out of therapy if they had lower verbal ability scores. From the standpoint of those delivering the interventions, mental health clinicians reported that lack of understanding of language disorders has led to feelings of “failing” this cohort (Hancock et al., 2023). Thus, adapted interventions for young people with DLD that consider their unique needs are essential to improve access to mental health support, outcomes, and therapist confidence.

This project aimed to bridge this gap by developing tailored materials for young people with DLD, designed for Educational Mental Health Practitioners (EMHPs) to deliver within school environments. EMHPs are graduate‐trained practitioners that work as part of mental health teams linked with schools and colleges, to support students with mild‐to‐moderate mental health difficulties. They are responsible for delivering evidence‐based, short‐term (4–8 sessions) interventions and helping to develop whole‐school mental health approaches (Ellins et al., 2021). We chose to focus on adapting EMHP intervention materials given the current expansion of EMHP roles across the UK, aimed at increasing access to mental health and wellbeing support in educational settings (Department of Health and Department for Education, 2017). The EMHP manuals are designed for neurotypical students, but given the large overlap between mental health referrals and language disorders (Cohen et al., 2013), most EMHPs frequently support students with DLD. Thus, by adapting materials that are being widely rolled out in schools, we can better meet the needs of students with DLD by equipping EMHPs with materials that allow them to work effectively with young people with DLD.

EMHPs are trained to use a workbook with their clients that provides psychoeducation information in written form alongside exercises for students to complete with support. In this project we chose to create videos to support delivery of the psychoeducation content. Video format was chosen for its ability to combine visual, auditory and textual components, which improves comprehension for those who may struggle with traditional “text‐based” materials. In a scoping review of psychoeducational interventions for people with complex communication needs, Watson et al. (2023) identified using multi‐media content and simultaneous visual cues to text as key strategies for improving accessibility for this population, which is easily facilitated through video‐based delivery. Additionally, videos can be accessed via multiple digital platforms including smartphones and computers, which widens their reach and accessibility. We chose to focus on the psychoeducation component of therapy, rather than the exercises, as this is the part that the EMHPs involved in our project identified as being more challenging to deliver with young people with communication challenges as it involves didactic teaching. Psychoeducation is a critical part of therapy as it provides the foundational information that is required to understand the purpose of exercises that follow, thus increasing treatment participation and satisfaction (Oliveira & Dias, 2023).

### Co‐production and young people with DLD

Co‐production is a type of collaborative process that aims to integrate key “stakeholders” into the research process, ensuring that their voices help shape the direction and potential outputs of a study. There is a growing body of literature advocating for co‐production in health interventions, as it supports the development of more effective, meaningful and inclusive solutions (Hassett et al., 2024). However, even with the widening acceptance and utilization of co‐production in research, many co‐production processes implicitly assume high language proficiency among participants. Taking McGeown et al.’s (2023) collaborative “love to read” project with teachers as an example, common features of co‐production include lengthy group meetings, time‐pressured feedback activities, and examining text‐heavy documents like research plans and program content, for example, Consequently, those with DLD are inadvertently excluded, or deterred, from fully participating.

This is problematic, as co‐production may be particularly important for populations whose voices are underrepresented or may struggle to self‐advocate, such as young people with DLD. Fletcher‐Watson et al. (2021) argued that researchers in fields such as Autism, ADHD and DLD have a “moral responsibility” to these more vulnerable groups to provide meaningful opportunities to shape the direction of research that will, ultimately, significantly affect their lives. For all involved, inclusive research may promote better quality of life, strengthen researcher‐collaborator relationships, and increase self‐esteem and self‐determination (Pellicano et al., 2014). This then leads to more valuable and applicable outcomes. Moreover, for groups of individuals that experience the world very differently, such as neurodivergent people including those with DLD, complete understanding and research development is not possible without the perspectives of those with lived experience (Fletcher‐Watson et al., 2021).

Despite the recognized benefits, there is a considerable lack of literature involving young people with DLD in co‐production or describing how to adapt the process for this population. Inspiration can be drawn from co‐production and co‐design methodologies employed with other neurodiverse populations. For example, Cullingham et al. (2024) described the experience‐based co‐design process of an anxiety intervention for children with autism. Monthly meetings had a pre‐sent informal agenda, used PowerPoint to share the content visually, and scheduled ample time for discussion. Processing breaks and check‐ins were given after intensive content was delivered. Similarly, Armitt et al. (2024) co‐produced a novel nature‐based intervention with children with ADHD, their families and professionals. Family and professional workshops were held separately to begin with, as the researchers felt that it would promote a more open discussion of experiences and perceived challenges. Workshops employed interactive tasks to keep the children engaged, and presented feedback visually using a graphic designer. Above all, both studies emphasized the importance of being reflexive when working with co‐producers with additional needs, allowing space to change to the “plan” if necessary. This not only maximizes the effectiveness of the collaboration but helps to build and maintain trust—something that the AASPIRE guidelines for including adults with autism in research also highlights (Nicholaidis et al., 2019).

While researchers aiming to carry out co‐production with young people with DLD can take inspiration from the literature on co‐production with other neurodiverse population, there are no specific studies or guidelines addressing co‐production with young people with DLD, representing a clear gap in the literature. To address this gap, this study describes how experienced‐based co‐design (EBCD) methodologies were adapted to enable young people with DLD to fully participate, and reports detailed findings from this process. This work is vital for understanding how best to involve young people with DLD in research and to ensure that their participation is meaningful.

## METHODS

The co‐design process was undertaken for a wider project aiming to test whether comprehension of psychoeducation materials can be improved by adapting delivery for young people with DLD. This project has been approved by the Research Ethics Committee at UCL (project number: 16571/002, date of approval: 01/07/2024). Here we describe the process to developing the adapted psychoeducation materials that will be used in an experimental study. We employed an Experience‐Based Co‐Design (EBCD) approach (Donetto et al., 2015) to develop a set of videos explaining key psychoeducational concepts in which the delivery is optimized for young people with DLD. EBCD was used to ensure that the experiences and insights of stakeholders ‐ in this case, young people with DLD, their families, and EMHPs ‐ directly influenced the design process. By centering these groups in the design process, EBCD ensures that any resulting materials and interventions are grounded in the real‐world experiences of those most affected. Thus, their diverse needs can be better met. Research has successfully used EBCD procedures to produce accessible mental health interventions for individuals with autism (Cullingham et al., 2024) and intellectual disability (Hewitt et al., 2025), but not DLD.

### Collaborating teams

1. ***Research Team***: The research team was responsible for designing the study, facilitating co‐design sessions and analyzing feedback. The team, based in the department of Clinical, Educational and Health Psychology at University College London, included a research assistant with a master's degree in psychology (IM), the lead investigator who is an academic psychologist (SG) and a co‐investigator who is an academic psychologist and trained Speech and Language Therapist (CN). None of the research team have language or communication difficulties.
2. ***Young Person and Parent Advisory Group***: The Young Person and Parent Advisory Group (YPAG) consisted of four adolescents (aged between 14 and 18) with lived experience of DLD. Three of the adolescents were accompanied by their parents who did not have DLD, who also acted as advisors.
3. ***Professional Advisory Group***: The Professional Advisory Group (PAG) consisted of four qualified EMHPs, a senior wellbeing practitioner, and an assistant psychologist—all with current or previous experience delivering the EMHP manuals for depression and anxiety.

All members of the Research team, YPAG and PAG were based in the UK.

### Stages of Co‐Production

**FIGURE 1:**
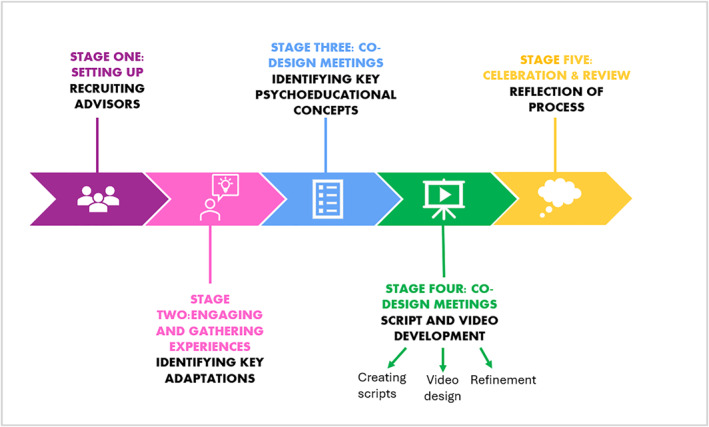
Stages of Co‐Production.

The Co‐Production process, in line with the stages of EBCD outlined in Donetto et al. (2015), is outlined in Figure 1 and described in detail below. The first four stages were conducted over a 6‐month period, and the reflection stage 3 months afterward.

1. ***STAGE 1*: *Setting up*: *Recruiting Advisors*.** Advisors were recruited via several platforms. For the YPAG, connections were made from a charity for speech and language difficulties (NAPLIC), and a flyer was posted on the charity Facebook page. One YPAG advisor was also an existing advocate for increasing awareness of DLD already known to the research group. For the PAG, all advisors were recruited by word of mouth through professional networks. All advisors expressed their interest via email and were given full information about the process in an accessible format and had the opportunity to ask any questions, before arranging a meeting date. Advisors provided verbal informed consent to take part in meetings and activities at each stage of the process.
2. ***STAGE 2*: *Engaging and gathering experiences*: *Identifying Key Adaptations*.** In addition to making introductions and hearing about their general experiences, early meetings explored what adaptations young people found most (and least) useful when talking to someone. Questions to facilitate this conversation included: “Did (therapist) do anything that made it hard for you to understand?“, “Did (therapist) do anything that was helpful for you to understand?” and “Can you think of anything else that is helpful for you?” Example responses were provided for the young people to endorse, along with the option to add further information, to make the task less open‐ended and offer clearer guidance.
  The PAG were also asked about key adaptations, but from the perspective of challenges they have faced delivering to young people with speech, language and communication needs, and any adaptations they made themselves. Questions to facilitate this conversation included: “Are there any elements of the session that you found particularly challenging?” and “Did you do anything differently when working with YP with communication difficulties?”
  The result of this stage was a list of recommendations for adapting therapy for young people with DLD.
3. ***STAGE 3*: *Co‐design meetings*: *Identifying Key Psychoeducational Concepts*.** The research team went through the EMHP manuals for depression and anxiety in detail, noting down any concepts that stood out as being important to learn. These were presented to the YPAG and PAG, along with the question “do you think any concepts are missing from the EMHP manuals that would be important for young people to understand?” (PAG) or “is there any other information you think would be important to include as a video?” (YPAG, directed at parents). This informed the selection of 10 key psychoeducational concepts to be adapted and developed into videos.
4. ***STAGE 4: Co‐design meetings: Script and Video Development.***
  - ***Creating Scripts*.** For each of the psychoeducational concepts identified in Stage 2, the paragraph explaining it was copied directly from the manual; becoming the “unadapted script.” Then, using the feedback from Stage 1, changes were made to the script to make it easier to understand, while maintaining the original meaning of the text. These became the “adapted scripts.” The scripts were iteratively presented to the advisory groups (both in online meetings and at‐home tasks) for feedback and revised as necessary.
  - ***Video Design*.** The advisory groups compared different platforms and styles for the videos to be created on—providing feedback on practicality, esthetic and personal preferences. Once decided, the scripts were added to the videos as voiceovers and iteratively presented to the advisory groups.
  - ***Refinement*.** After several rounds of feedback and refining the videos, the final versions were created.
5. ***STAGE 5*: *Celebration and review*: *Reflection of Process*.** All advisors were sent an anonymous evaluation survey to complete online, allowing them to reflect on how they found the co‐production process. The survey included questions about collaboration and communication, the meetings and offline tasks, the quality and appropriateness of the videos produced, and any final thoughts. Advisors could feed back via ratings (e.g., “Bad/Okay/Good/Great”) and (optional) open‐text responses with prompts for elaboration.

### Meeting structure

When the research team recruited the YPAG, they asked whether members would like to have meetings online or in person, and whether they would like individual meetings or group meetings. This consideration took into account that DLD can make contributing to group sessions more challenging, particularly when interacting with unfamiliar peers. Adolescents with DLD have reported a preference for talking in small groups where they feel secure (Ekström et al., 2023). The majority expressed a preference for meetings to be conducted one‐on‐one and online. The YPAG meetings were therefore carried out one‐to‐one online whereas the PAG meetings were carried out in a group online. Advisors were compensated for their time with Amazon or Love2Shop vouchers, at a rate of £25 an hour. For both groups we employed a combination of live meetings and offline feedback opportunities to aid engagement and ensured that everyone could contribute meaningfully in a way that best suited their needs. In addition, the flexible format allowed for varying levels of parental involvement in the YPAG sessions, recognizing that parents or caregivers of young people with speech and language needs can provide communication support and act as advocates (Davies et al., 2017). Several other accommodations were made to ensure that the meetings were accessible:

#### Pre‐meeting preparation

**FIGURE 2:**
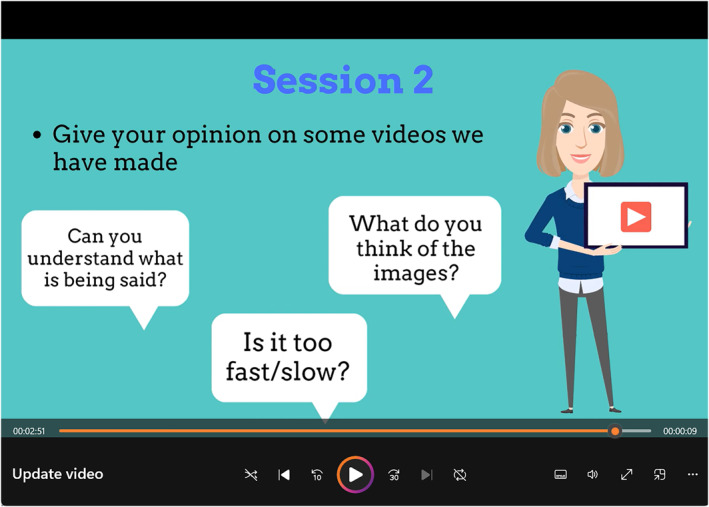
Screenshot from one of the pre‐meeting information videos sent to advisors.

Meeting schedules were sent out in advance, containing a summary of the key points and questions that would be discussed. This was sometimes accompanied by a video explaining the key information in a visual format (See Figure 2 for an example). Given that people with DLD may struggle with real‐time word retrieval and encoding information (McGregor et al., 2017), this allows advisors to familiarize themselves with key vocabulary and rehearse questions in advance. This facilitates deeper confidence and engagement during the actual meeting.

#### In‐meeting facilitation

**FIGURE 3:**
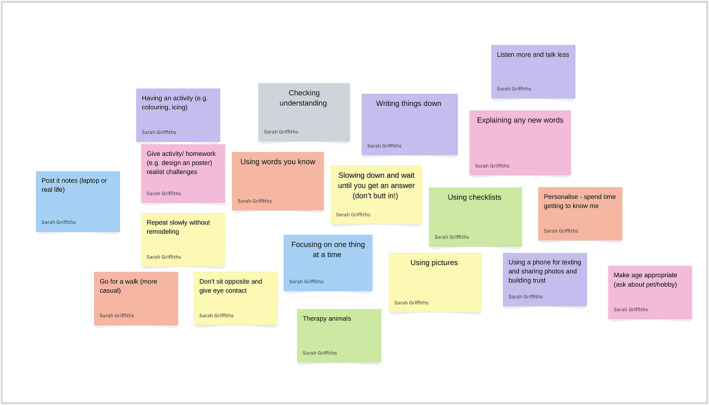
One of the mind maps generated in a meeting with a member of the YPAG. Titled “What changes should therapists make when talking to you?”.

During the meetings themselves, a variety of tools and techniques were employed to optimize participation and communication. Visual aids such as post‐it notes and mind maps were shared and updated during discussions (See Figure 3 for an example). Individuals with language disorders experience increased cognitive load from language processing, therefore providing visual cues in real‐time to support language processing can reduce cognitive demands (Washington & Warr‐Leeper, 2013) It also slowed the pace of the conversation, as each point had to be recorded on the visual aid before the conversation moved on, aligning with slower processing speeds in DLD across verbal and non‐verbal domains (Zapparrata et al., 2023) The researchers aimed to use simplified language both in their written records and verbal conversation to support accessibility. Open questions were avoided as they place greater demands of expressive language skills and provide limited retrieval cues (Graesser et al., 2010), providing instead a list of options for endorsement, with space for additional ideas if the young person chose. Moreover, flexible response options, including verbal responses, written comments, and a star‐based rating system were offered to advisors in the offline tasks. This meant they could contribute in the way that felt most comfortable to them.

#### Post‐meeting reflection

**FIGURE 4:**
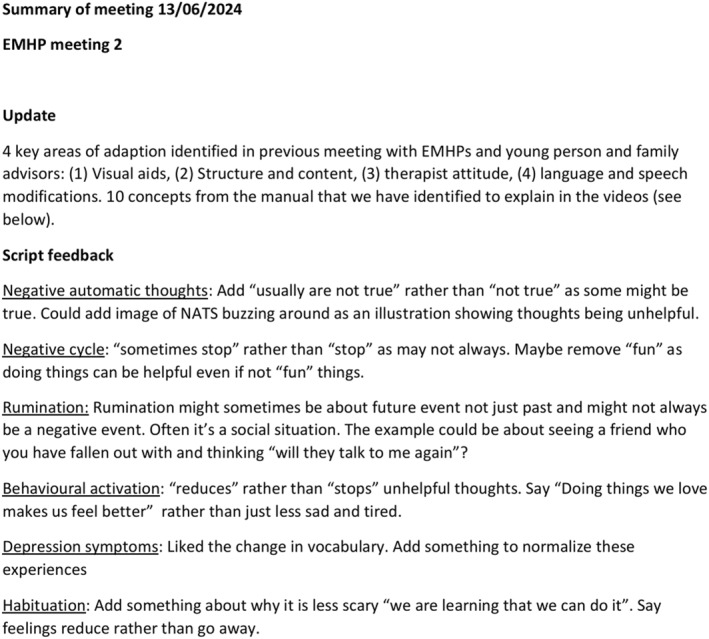
One of the post‐meeting summaries sent to the PAG.

After each meeting, the researchers shared a written summary of the meeting with all participants. This provided an opportunity for reflection and processing at their own pace and allowed the opportunity to identify and correct any miscommunication (See Figure 4 for an example).

### Collating and analyzing feedback

Meeting contributions were documented through facilitator notes (IM) and participant‐generated mind maps and sticky notes. These materials were collated and cross‐checked with advisors to ensure completeness, before being reviewed in full and synthesized into key themes and take‐away points, and visual summaries. Task responses submitted via Qualtrics were reviewed and summarized into key points, which were then discussed in subsequent meetings.

Evaluation survey responses were collected using the platform Qualtrics. Quantitative items were summarized descriptively. Open‐text responses were read in full and summarized using a surface‐level content analysis, with key ideas grouped into themes.

## RESULTS

### Key insights

#### Recommendations for adapting therapy

**FIGURE 5:**
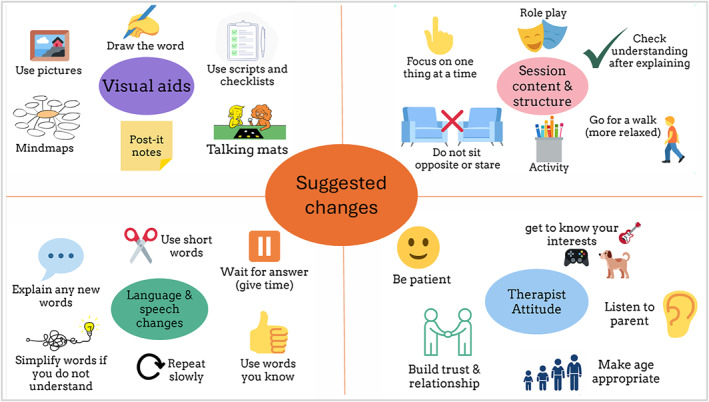
Recommendations for adapting therapy for young people with developmental language disorder.

Stage Two of the process produced a comprehensive list of recommendations for adapting therapy for young people with DLD. This is summarized in Figure 5 across four key categories: visual aids, session content and structure, speech and language changes, and therapist attitudes. Our focus on creating psychoeducation video resources limited our ability to apply all the recommendations (e.g., going for a walk or getting to know interests), but it still provided valuable insight into what young people with DLD want in therapy that can inform future research and practice guidelines.

The adaptations which we were able to implement in creating the video content were as follows:

*Slowing down speech and content.* Young people with DLD reported that slowing down both speech delivery and pacing of information helps them absorb information and engage more effectively. This also includes factoring in more pauses to allow adequate time for processing.

*Using visual aids.* The use of visual supports such as images, mind maps, and written keywords is highly beneficial in reinforcing spoken content, according to the YPAG. The PAG also reported trying to make their resources more visual when working with young people with speech, language and communication needs.

*Simplifying complex language.* Simplifying complex vocabulary is key to ensuring that the (often abstract) therapeutic content is more comprehensible. In addition, using shorter words and words known to the young person.

In contrast, things that made it hard to engage in therapy included using complex vocabulary and abstract concepts, long passages of speech with no visual accompaniments or pauses, and worksheets with lots of text.

#### Recommendations for psychoeducational scripts

In general, the PAG thought that the scripts were simplified well and still included key information, although minor suggestions were made. For example, not removing key terminology like “negative thoughts”, keeping terminology the same throughout, and avoiding saying that symptoms will go away completely.

The YPAG identified one main area as requiring more change:

*Including concrete examples.* Some of the young people with DLD reported struggling to understand the concepts described in the video without the use of a concrete example—for example, “Anxiety Habituation”. As a result, we ensured each video had a clear example that they could relate to—for example, “we might be scared of starting a new school. On the first day, we feel more and more scared, but after a few days we start to feel better.”

*Recommendations for psychoeducational videos.* The overall response to the videos was very positive, with the PAG reporting that they liked the format of the videos and the accurate visual representation of the concepts. The YPAG valued the colorful visuals, simplicity and slower pace of the language. Two areas were identified as requiring change by the YPAG:

*Female‐only voiceover.* While the voiceover was generally well‐received, it was suggested that including a male voice could better reflect the diversity of young people viewing the videos. As a result, we re‐recorded half of the videos with a male voice, ensuring that the tone and delivery was as close to the original as possible.

*Ambiguous visuals.* A small number of the visuals were not explicit enough for the YPAG, which caused confusion. For example, one video contained a lightbulb in it to represent the brain being activated, which was too abstract. In another, the facial expression of the character was misconstrued as boredom. As a result, we re‐designed such images to remove any ambiguity.

#### Advisory group reflections

Four members of the PAG (out of six) and three young people in the YPAG (out of four) completed the anonymous evaluation survey, 3 months after finalization of the videos.

*Communication.* Both the YPAG and PAG consistently highlighted the strength of communication with the research team, and felt that their opinions and ideas were listened to either “always” or “most of the time.” This sense of being heard fostered a collaborative atmosphere, with one PAG member noting: “The researchers explained everything well and welcomed all feedback discussed in sessions.”

*Meetings and tasks.* Both groups liked the structure and content of the meetings, particularly the informational videos that were shared prior to each session, which helped prepare them for discussions. However, one young person pointed out that “Zoom meetings can be hard sometimes because of lags and worrying about time limits.” The asynchronous tasks were generally viewed positively, especially by the PAG, who valued the flexibility these tasks offered in accommodating their schedules. That said, some of the YPAG found the longer asynchronous tasks more difficult, particularly ones with more to get through: “Sometimes it was tiring ‘cos of all the information. Sometimes I had to watch scenarios a few times to understand what I was being asked.”

*Videos produced.* There was unanimous enthusiasm for the co‐produced videos among both groups. All advisors rated the videos as highly important, highly relevant, and appropriately adapted for individuals with DLD. Young people felt that they would help others like them to better understand their mood and feelings. Likewise, the PAG thought the videos would be welcomed by other mental health practitioners delivering low‐intensity content.

*Co‐production role and process.* Young people reported that they enjoyed their role as advisors, valuing the opportunity to express their views and be heard. One young person shared, “I liked saying what I want,” while another reflected on the importance of advocacy, saying, “I wanted to make a difference ‘cos living with DLD is so hard and frustrating.” They also felt like they learned something from being involved. Similarly, the PAG felt it was a very positive experience that supported their learning and development—with one stating “Collaboration (with other advisors) helps to identify what type of language is used among different young people.”

## DISCUSSION

### Researcher reflections

The collaborative process proved to be highly successful in creating a richer and more relevant set of psychoeducational videos for young people with DLD. Through regular feedback, we gained valuable insights into content, language, and visuals; uncovering elements that would have otherwise been overlooked. For example, advisors suggested avoiding certain phrases that were deemed unhelpful—for example, suggesting that anxiety will go away completely (when it is better to say that you will get more confident or it will get less scary), and using concrete examples.

The recommendations of the advisor's map onto what is known about the nature of communication challenges in DLD. For example, the recommendation to link abstract concepts to familiar scenarios helps to ground new concepts in existing knowledge, thereby reducing cognitive load and supporting learning (Micallef & Newton, 2022). Similarly, using clear, unambiguous visuals minimizes the risk of misinterpreting visual input, as individuals with DLD can be over‐literal in their comprehension (Bishop et al., 2017). Clear understanding is particularly important in psychoeducation as it forms the basis of each therapy module (Oliveira & Dias, 2023).

This collaboration also highlighted the importance of working with the preferences of the advisor. By varying the format of feedback options, and giving advisors the autonomy to choose this, we maximized engagement. We also built strong rapport and trust with the advisors, fostering a more effective and co‐operative working relationship. This is important for all co‐production processes but particularly in the context of co‐production with young people with DLD who report fewer communication challenges when they feel at ease with their conversation partner (Ekström et al., 2023), thus allowing them to effectively participate in the co‐production process despite their communication challenges.

However, the process also presented several challenges. One notable issue was receiving conflicting feedback from advisors, such as differing opinions on the relatability of the examples, the amount of text on pages, and the choice of certain language. In these cases, we had to prioritize the majority's preferences and carefully explain the rationale behind our decisions. Additionally, some feedback sessions or tasks turned out to be too long for young people with DLD, which led to disengagement by the end. This resulted in less informative responses due to fatigue. Furthermore, conducting individual meetings made it harder to foster a collective vision and make joint decisions, which slowed the process down. That said, a group setting would have been inappropriate for some of the YPAG, therefore individual meetings were deemed as the better option. Finally, the use of prompts rather than open‐ended questions also presented a challenge, as it may have influenced the opinions shared. This makes it harder for the YPAG to have an equal influence. However, this concern needs to be balanced against the benefits of using prompts, which helped make it easier for young people with DLD to contribute.

It should also be noted that our advisors were not involved at every stage of the project, notably the early stages of research question and design formulation. While they were paid for their involvement, this was at an hourly rate, approximately once a month, during a 6‐month period. As a result, their input was more limited compared to the ongoing work of the researchers, and the final decisions rested with the core research team. This reduces the level of full and equal collaboration. Future research should explore ways of involving young people with DLD as advisors from inception, or even as co‐researchers—something that has been demonstrated in the Love to Read project, for example, (McGeown et al., 2023).

### The role of parents in Co‐production with young people with DLD

Another important thing to consider when working with this population is the position of the parent. Caregiver support and engagement in collaborative environments such as therapy is often critical to its success for the child—“the family is the essential environment in which children grow and develop ‐ whatever its composition.” (Rosenbaum, 2016). This may be especially true for young people with DLD, whereby caregivers often provide extra language and communication support, advocate for the young person, help with social challenges, and build confidence (Klatte et al., 2024). Indeed, in the current project, only one of the advisors chose to take part without the presence of their parent or caregiver. Involvement of parents is positive in terms of encouraging the young people to give their views and support them with their specific communication needs. However, it may also be a limitation in that parental views may conflict with, or inadvertently influence, the young person's views. Several studies in the autism field, for example, report a disconnect between views and expectations of parents of people with autism, and the individuals themselves (Cheak‐Zamora, 2015; Kirby et al., 2002). In the context of co‐production, it is therefore important to recognize (and utilize) the role of the parent while also clearly distinguishing between their perspectives and those of their child with DLD.

### Implications and future research

The outcome of this project has several important implications for future research. First, while the co‐production process was successful in developing adapted psychoeducational videos for young people with DLD, the insights gained from our discussions with the YPAG extended beyond the scope of the current project. The recommendations we were able to apply in Phase 2 were specific to research practices and EMHP manuals (e.g., simplifying language and using visuals), whereas it was clear that wider, service‐level changes were also required. Future studies could explore the broader range of changes and interventions that we identified, particularly those involving other stakeholders such as schools, parents, and healthcare providers. Additionally, while the co‐produced videos were well‐received by the advisory groups, they have yet to be tested in real‐world settings. The next logical step in this line of research will be to evaluate the effectiveness of the videos in improving understanding of psychoeducational concepts, and improving mental health outcomes for young people with DLD.

Finally, although this project focused specifically on young people with DLD, many of the recommendations may have broader relevance for other populations with language difficulties and neurodivergence, who are also at risk of poorer mental health outcomes. This study highlights the importance of accessible communication and meaningful participation in research and co‐design, regardless of specific diagnostic group.

## CONCLUSIONS

To conclude, this study demonstrates how young people with DLD can be effectively integrated into the research process, acting as a useful reference for others in the field. This approach could lead to more relevant and impactful research and interventions for the DLD population, allowing them to have a voice. However, we must consider the variability of communication preferences, higher susceptibility to influence (i.e., from prompts or parental/researcher opinion) and the additional time and resources required to maximize accessibility (such as having longer deadlines and creating infographics and videos). Future research should work flexibly within this methodology to engage those with DLD, as well as wider populations with language needs, in the process.

## AUTHOR CONTRIBUTIONS

**Isabella Metcalfe**: Conceptualization; investigation; visualization; methodology; writing—original draft. **Courtenay Norbury**: Conceptualization; methodology; writing—review and editing. **The DLD Young Person and Professionals Advisory Group**: Conceptualization; investigation; writing—review and editing. **Sarah Griffiths**: Conceptualization; investigation; methodology; funding acquisition; writing—review and editing.

## CONFLICT OF INTEREST STATEMENT

The authors declare no conflicts of interest.

## ETHICAL CONSIDERATIONS

All members of the young person advisory group provided verbal informed consent at each stage of the project and have contributed to and approved the publication of this manuscript as co‐authors. All of the professional advisory group provided verbal informed consent at each stage of the project, and 3 out of 6 contributed to and approved the publication of this manuscript as co‐authors. Ethical approval for developing and testing adapted CBT materials was provided by the Research Ethics Committee at UCL (project number: 16571/002, date of approval: 01/07/2024). Ethical approval was not required for the co‐production element of this project as it did not involve collection of data from research participants.

## Data Availability

Data sharing not applicable to this article as no datasets were generated or analyzed during the current study.
